# Non-invasive myocardial work index contributes to early identification of impaired left ventricular myocardial function in uremic patients with preserved left ventricular ejection fraction

**DOI:** 10.1186/s12938-022-01023-5

**Published:** 2022-08-13

**Authors:** Huizhen Zhu, Yanan Li, Cunying Cui, Danqing Huang, Yuanyuan Liu, Ruijie Liu, Qingqing Zhao, Ying Wang, Lin Liu

**Affiliations:** Department of Ultrasound, Fuwai Central China Cardiovascular Hospital, Henan Provincial People’s Hospital, Central China Fuwai Hospital of Zhengzhou University, People’s Hospital of Zhengzhou University, No.1, Fuwai Road, Zhengzhou, 451464 Henan China

**Keywords:** Echocardiography, Uremia, Hemodialysis, Myocardial work, Ventricular function, Left

## Abstract

**Background:**

Cardiac damage is the leading cause of death in uremic patients. This study aimed to evaluate the application of non-invasive myocardial work index (NIMWI) by echocardiography in assessing the left ventricular (LV) systolic function in uremic patients.

**Methods:**

Twenty-six uremic patients and 27 age- and sex-matched healthy volunteers were enrolled in the study. Except for the conventional echocardiographic parameters, the LV myocardial work (MW) parameters including GWI (myocardial global work index), GCW (global constructive work), GWW (global wasted work), and GWE (global work efficiency) were calculated in study participants. Differences in MW parameters between the uremic and normal groups were compared by independent-sample *t*-test. Receiver operating characteristic (ROC) curves were constructed for MW parameters to detect abnormal LV systolic function in uremic patients.

**Results:**

Compared with the normal group, GWW was significantly increased and GWE decreased in the uremic group (*P* < 0.05). Area under the curve (AUC) for GWE by the ROC analysis was 0.966. The best threshold, sensitivity and specificity values of GWE to detect abnormality of LV systolic function in uremic patients were 92.5%, 0.89 and 0.96, respectively.

**Conclusions:**

NIMWI may be applied to assess the global MW of uremic patients. The presence of reduced GWE can help identify impaired left ventricular myocardial function in uremic patients with preserved LV ejection fraction with a high sensitivity and specificity.

## Background

Chronic kidney disease (CKD) is a common disease which seriously threatens patients’ health and life. The disease causes damage to multiple systems and organs, especially in its end-stage (i.e. uremia). Indeed, cardiac damage is the leading cause of death in uremic patients [1]. Earlier studies have demonstrated that myocardial contraction function of the uremic patients has been impaired when the left ventricular ejection fraction (LVEF) is normal [2]. Therefore, an objective and accurate method to evaluate left ventricular (LV) systolic function in uremic patients is urgently needed.

The pressure–volume loop method measured by cardiac catheterization can assess the LV systolic function and myocardial oxygen consumption accurately [3]. However, this method is fairly invasive and has limited application in clinical practice. LVEF, measured by conventional transthoracic echocardiography, is the indicator most commonly used to predict mortality or morbidity of cardiovascular disease; Nonetheless it is often influenced by pre- and post-loads and the cardiac structure [4]. Previous studies reported that the global longitudinal strain (GLS) obtained by two-dimensional (2D) speckle tracking technology is more sensitive for diagnosing abnormalities of LV systolic function than conventional LVEF [5]. However, myocardial strain is susceptible to the cardiac afterload, which can affect the accuracy of myocardial function evaluation. The non-invasive myocardial work index (NIMWI) is a new method based on 2D speckle tracking technology that combines myocardial deformation and LV pressure to evaluate LV myocardial work (MW), which is more objective than GLS alone [6]. Russell et al. demonstrated a good correlation between NIMWI assessment and invasive and direct measurements of MW [7].

Previous studies have indicated that the NIMWI can evaluate the LV MW in patients with CKD without dialysis [8], but the MW in CKD patients undergoing haemodialysis treatment has not been studied. Thus, this study aimed to evaluate the feasibility of the NIMWI for assessing LV global MW in uremic patients with preserved ejection fraction undergoing haemodialysis, and provided reference values for the clinical evaluation of LV systolic function in these patients.

## Results

### Clinical characteristics

Compared with the normal group, the uremic group showed higher systolic blood pressures (SBP) and diastolic blood pressures (DBP). The differences between groups were statistically significant (both *P* < 0.05). There were no differences in gender, age, heart rate (HR) and body surface area (BSA) between the two groups (*P* > 0.05) (Table 1).

**Table 1:** Comparison of general clinical data in the study groups

Variable	Normal group *n* = 27	Uremic group *n* = 26		
			*χ*^2^	*P*-value
Male/female	13/14	13/13	3.000	0.083
			*t*	*P*-value
Age (years)	44.0 ± 79.34	49.11 ± 10.56	1.842	0.071
BSA (m^2^)	1.72 ± 0.16	1.75 ± 0.18	0.732	0.468
HR (times.min^−1^)	69.0 ± 06.87	72.84 ± 10.76	0.732	0.130
SBP (mmHg)	120.37 ± 8.49	157.50 ± 16.30^a^	10.456	< 0.001
DBP (mmHg)	78.59 ± 6.16	98.19 ± 12.07^a^	7.484	< 0.001

Values are mean ± standard deviation

*BSA* body surface area, *HR* heart rate, *SBP* systolic blood pressure, *DBP* diastolic blood pressure

^a^*p* < 0.05 vs. normal group, 1 mmHg = 0.133 kPa

### Traditional echocardiographic measurement

Compared with the normal group, the uremic group showed increased interventricular septum end-diastolic thickness (IVSTd), LV posterior wall end-diastolic thickness (LVPWTd), LV end-diastolic diameter (LVEDd), LV mass index (LVMI) and decreased GLS. The differences between groups were statistically significant (*P* < 0.05). There were no significant differences in LVEF between the two groups (*P* > 0.05) (Table 2).

**Table 2:** Conventional echocardiography parameters in the study groups

Variable	Normal group *n* = 27	Uremic group *n* = 26	*t*	*P*-value
IVSTd (mm)	8.94 ± 0.81	11.96 ± 1.24^a^	10.461	< 0.001
LVPWTd (mm)	8.96 ± 0.75	12.00 ± 1.29^a^	10.360	< 0.001
LVEDd (mm)	43.33 ± 3.92	50.69 ± 6.71^a^	4.848	< 0.001
LVEF (%)	64.29 ± 2.05	62.61 ± 4.38	− 1.777	0.084
LVMI (g/m^2^)	73.02 ± 13.86	138.10 ± 34.40^a^	8.965	< 0.001
GLS (%)	− 18.77 ± 0.93	− 14.11 ± 2.26^a^	9.717	< 0.001

Values are mean ± standard deviation

*IVSTd* interventricular septum end-diastolic thickness, *LVPWTd* left ventricular posterior wall end-diastolic thickness, *LVEDd* left ventricular end-diastolic diameter, *LVEF* left ventricular ejection fraction, *LVMI* left ventricular mass index, *GLS* global longitudinal strain

^a^*p* < 0.05 vs. normal group

### Comparison of MW parameters

**Fig. 1:**
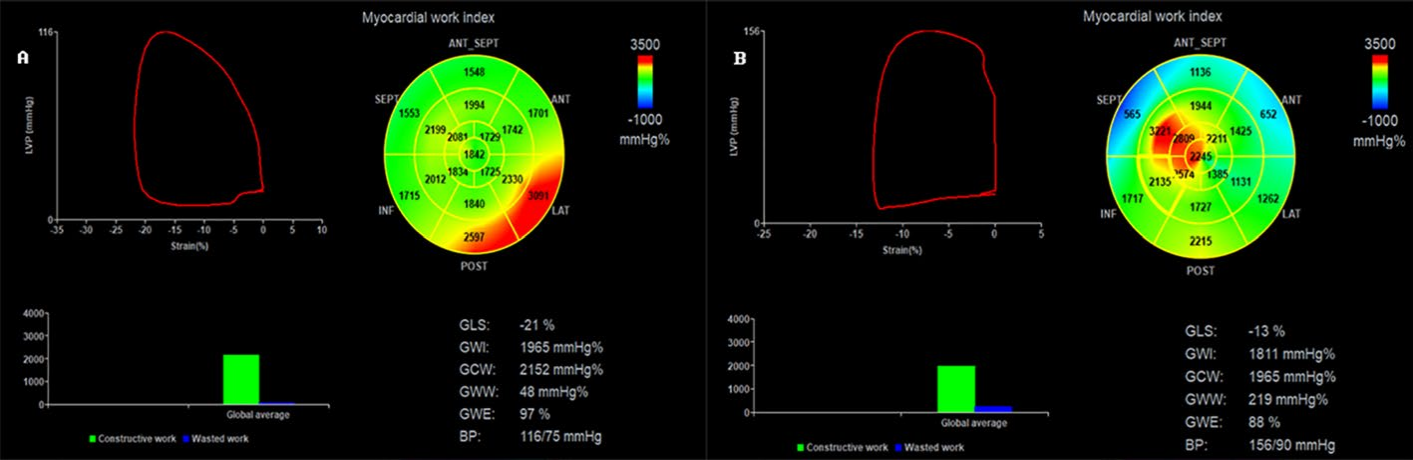
**A**: Left ventricular MW parameters of the normal group. **B**: Left ventricular MW parameters of uremic patients. In each group, the LV PSL, 17-segment bull's-eye representation of GWI, and the MW parameters were given, and the MW parameters included GWI, GCW, GWW, and GWE. MW: myocardial work; PSL: pressure–strain loops; GLS: global longitudinal strain; GWI: global work index; GCW: global constructive work; GWW: global waste work; GWE: global work efficiency

Compared with the normal group, the uremic group showed increased global wasted work (GWW) and decreased global work efficiency (GWE). The differences observed between groups were statistically significant (*P* < 0.05). However, differences in global work index (GWI) and global constructive work (GCW) between the two groups were not statistically significant (*P* > 0.05) (Table 3, Fig. 1).

**Table 3:** Comparisons of MW parameters in the study groups

Variable	Normal group *n* = 27	Uremic group *n* = 26	*t*	*P-value*
GWI (mmHg%)	1927.74 ± 164.01	1863.23 ± 284.71	− 1.006	0.321
GCW (mmHg%)	2086.55 ± 199.59	2163.26 ± 324.98	1.040	0.309
GWW (mmHg%)	83.77 ± 37.42	238.34 ± 1 ± 14.89^a^	6.637	< 0.001
GWE (%)	95.48 ± 1.80	88.80 ± 3.08^a^	− 9.655	< 0.001

Values are mean ± standard deviation

*GWI* global work index, *GCW* global constructive work, *GWW* global wasted work, *GWE* global work efficiency

^a^*P* < 0.05 vs. normal group

### Main relations of NIMWI

GWE and GWW showed a good correlation with SBP (*r* = − 0.83, *P* < 0.001 and *r* = 0.72, *P* < 0.001, respectively).

### Receiver operating characteristic (ROC) curve analysis

**Fig. 2:**
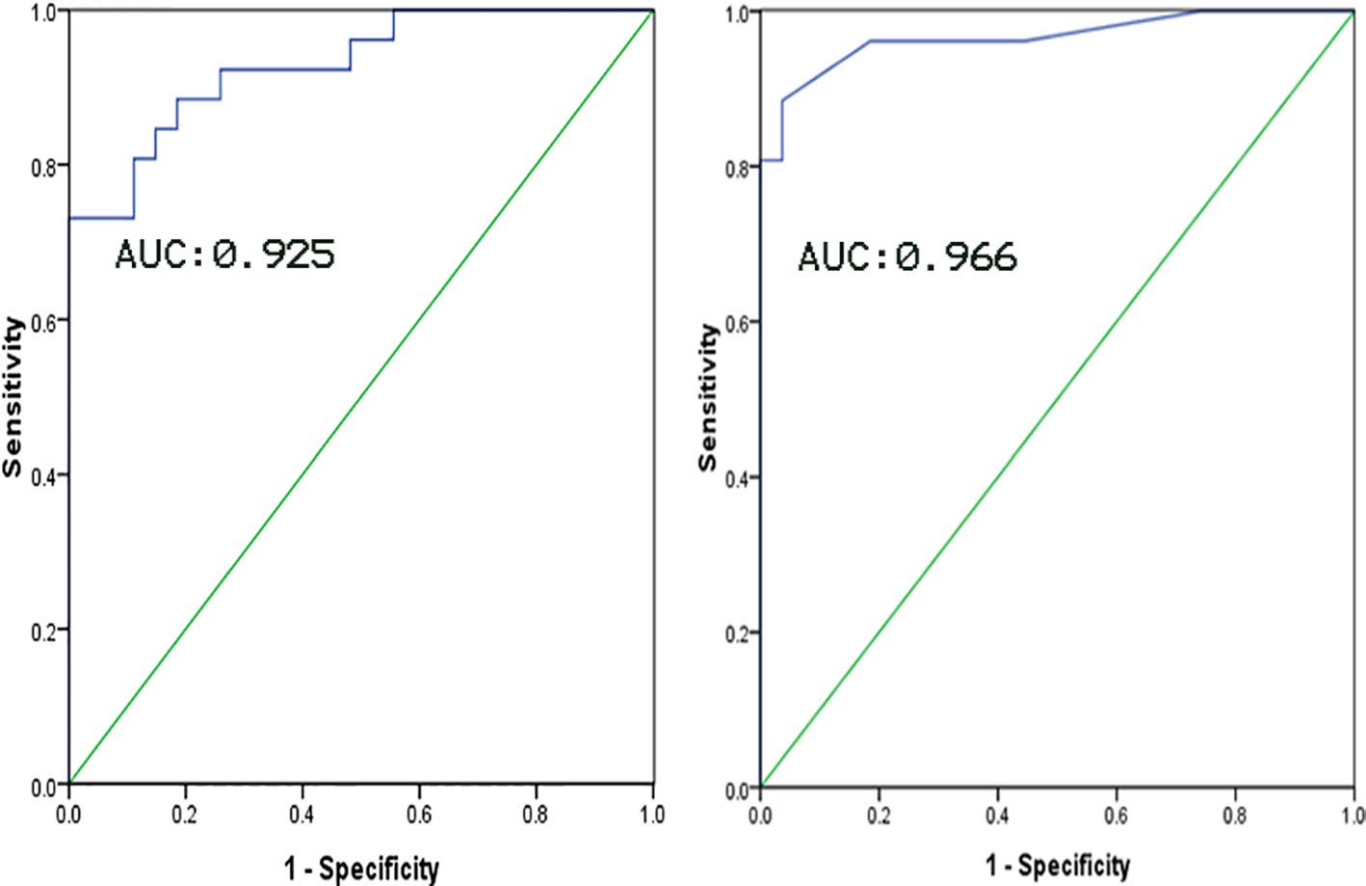
ROC curve analysis of the main MW parameters for identifying impaired LV myocardial function in uremic patients with preserved LV ejection fraction. On the left: ROC curve of GWW parameter (AUC = 0.925). On the right: ROC curve of GWE parameter (AUC = 0.966). GWE had better AUC than GWW with the sensitivity, specificity and optimal threshold of 89%, 96% and 92.5%, respectively. ROC: receiver operating characteristic; GWE: global work efficiency

Based on ROC curve analysis of main MW parameter, area under the curve (AUC) of GWE and GWW were 0.966 and 0.925, respectively. GWE was the best diagnostic index for identifying impaired LV myocardial function in uremic patients with preserved LV ejection fraction. The sensitivity and specificity were 89% and 96%, respectively. The best threshold of GWE was 92.5% (Fig. 2).

### Intra- and inter-observer variability

Intra-class correlation coefficients indicated that GWI, GCW, GWW and GWE between the intra- and inter-observer measurements were consistent (Table 4).

**Table 4:** Intra‑ and inter‑observer variability

Variable	Intra-observer ICC		Inter-observer ICC	
	ICC	95% CI	ICC	95% CI
GWI	0.995	0.979–0.999	0.992	0.970–0.998
GCW	0.961	0.843–0.990	0.993	0.970–0.998
GWW	0.961	0.842–0.990	0.972	0.889–0.993
GWE	0.983	0.932–0.996	0.971	0.883–0.993

*GWI* global work index, *GCW* global constructive work, *GWW* global wasted work, *GWE* global work efficiency,* ICC* intraclass correlation coefficients,* CI* confidence interval

## Discussion

Uremia is the final stage of CKD progression. Serious damage to the kidney functions promotes the production and accumulation of toxins in the body, resulting in multi-organ dysfunction and poor health. Cardiovascular complications are the main cause of death in patients with uremia [9]. Therefore, early detection of changes in myocardial contraction in uremic patients is important to enable the early control of cardiovascular complications. The results of our study showed that compared with the control group, there were no change in LVEF in uremic patients (*P* > 0.05), but the uremic group showed increased GWW and decreased GWE (*P* < 0.05), indicating that the NIMWI can identify LV systolic dysfunction in uremic patients with preserved ejection fraction. GWE was the best diagnostic index for identifying impaired LV myocardial function in uremic patients with preserved LV ejection fraction, and its sensitivity, specificity and optimal threshold were 89%, 96% and 92.5%, respectively.

Traditional evaluations of MW mainly rely on invasive pressure measurement, the feasibility of which is limited in conventional clinical practice [10, 11]. The results of MW index assessment of MW have a strong correlation with the measurement results of invasive cardiac catheterization [12]. This finding confirms the feasibility of the NIMWI for assessing MW. The NIMWI based on 2D speckle tracking technology, which combines myocardial deformation and non-invasive LV pressure to assess MW [7], is more comprehensive than GLS for the assessment of cardiac function in patients with altered afterload, and the technique can detect impaired myocardial function early, and its feasibility has been established in hypertension, hypertrophic cardiomyopathy, dilated cardiomyopathy and cardiac resynchronization [13–16].

Compared with the normal group, there were no difference in GWI and GCW, but GWW increased and GWE decreased in the uremic group, indicating that a decreased GWE of the left ventricle in uremic patients. GWE is the ratio of GCW to the sum of GCW and GWW, and there is no obvious change in the GCW. Thus, it can be seen that the decrease of GWE is mainly related to the increase of LV GWW. GWW refers to the work done by the left ventricle that is not conducive to ejection, including the total work done by cardinal elongation at systole and cardinal shortening at isovolumic diastole. Any factor affecting myocardial elongation at systole and myocardial shortening at isovolumic diastole may increase GWW. An earlier study [17] demonstrated that several factors in the body of with uremic patients, such as sodium and water retention and renin–angiotensin–aldosterone system activation, participate in the development of hypertension. The presence of long-term high loads leads to increased LV myocardial stiffness, ventricular remodelling, and myocardial oxygen consumption [18, 19]. Luca et al. [20] found that secondary nephritic anaemia in uremic patients aggravates cardinal ischaemia, hypoxia, damages cardinal cells by calcium and phosphorus imbalance, and causes microvascular ischaemia, interstitial fibrosis and cardiac myocyte hypertrophy by long-term haemodialysis-related myocardial stunning. These phenomena negatively affect myocardial contractions, resulting in an increase in global wasted work (GWW), and a decrease in the global work efficiency of the left ventricle.

In this study, GWE and GWW showed a good correlation with SBP, which is consistent with the rationale that the NIMWI technique evaluates myocardial work based on stress and strain. AUC for GWE by the ROC analysis was 0.966, which indicates that the GWE accurately evaluated LV systolic function in uremic patients. The best threshold, sensitivity and specificity values of GWE to detect abnormality of LV systolic function in uremic patients were 92.5%, 0.89 and 0.96, respectively. It can provide some reference values for the early detection of the LV systolic dysfunction in uremic patients with preserved left ventricular ejection fraction..

However, this study had some limitations that should be noted. It was a single-centre study with a relatively small sample size and no long-term follow-up of uremic patients, which will be encompassed in the next phase of the study.

## Conclusions

NIMWI may be applied to assess the global MW of uremic patients. AUC, sensitivity, and specificity for GWE parameter by ROC analysis were high, revealing the accuracy of GWE for the detection of the LV systolic dysfunction in the uremic patients. The reproducibility of MW parameters can help to improve its application in clinical practice.

## Methods

### Study population

A total of 26 uremic patients who were treated in the nephrology department of our hospital from November 2020 to June 2021 were consecutively identified. All uremic patients were treated with regular haemodialysis three times a week and with LVEF ≥ 50% according to the 2016 European Society of Cardiology Guidelines [21]. Patients were excluded if there were valvular disease, LV outflow tract obstruction, arrhythmia, essential hypertension, diabetes, previous myocardial infarction, medium and large pericardial effusion, LV systolic dysfunction with LVEF < 50%, and patients with a poor acoustic window of transthoracic echocardiography, which was mainly manifested as unclear display of endocardium or endocardium located outside the sampling frame.

Twenty-seven healthy volunteers matched for sex, age and body surface area (BSA) were selected as the normal group. All volunteers had no history of heart disease, hypertension, diabetes or medication. Their physical examination, electrocardiogram, chest radiograph, renal function and echocardiography results were normal.

**Fig. 3:**
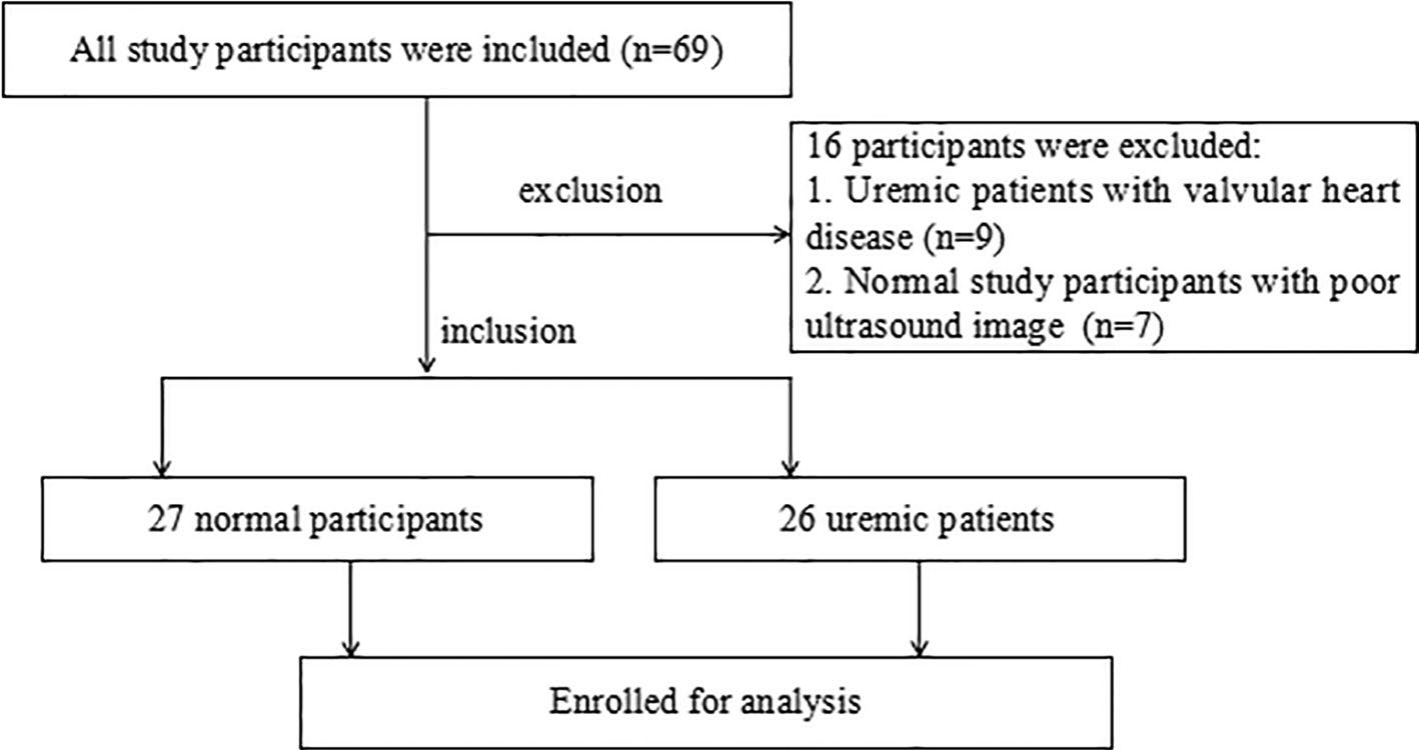
Flowchart of study populations

All subjects provided informed consent prior to participating in this study, and the study protocols were approved by the Hospital Ethics Committee. The participant selection process is illustrated in the flowchart in Fig. 3.

### Echocardiography

GE Vivid E95 (GE Vingmed Ultrasound AS, Horten, Norway) colour Doppler ultrasound system equipped with M5Sc-D 1.4–4.6 MHz probe was used for echocardiographic image acquisition.

The subjects were instructed to assume the left-lateral position and an electrocardiogram was attached. Transthoracic echocardiography was used to measure the LVEDd, LVPWTd and IVSTd of the participants. The modified biplane Simpson method was used to calculate LVEF from the apical four- and two-chamber views. The LVMI was calculated as LV mass divided by BSA. We obtained the dynamic two-dimensional images from the LV apical four-, two- and three-chamber views at frame rate of 58—69 frames/s (average of 67.4 ± 6 frames/sec) for at least three cardiac cycles in the resting state. The images were copied, saved and exported to the workstation in Digital Imaging and Communication in Medicine (DICOM) format on a mobile hard disk for offline analysis.

All two-dimensional images and measurements were performed according to American Society of Echocardiography guideline [22], and all parameters were averaged over three consecutive cardiac cycles. The SBP and DBP of the participants were measured using a brachial artery cuff.

### Two-dimensional speckle tracking image analysis

**Fig. 4:**
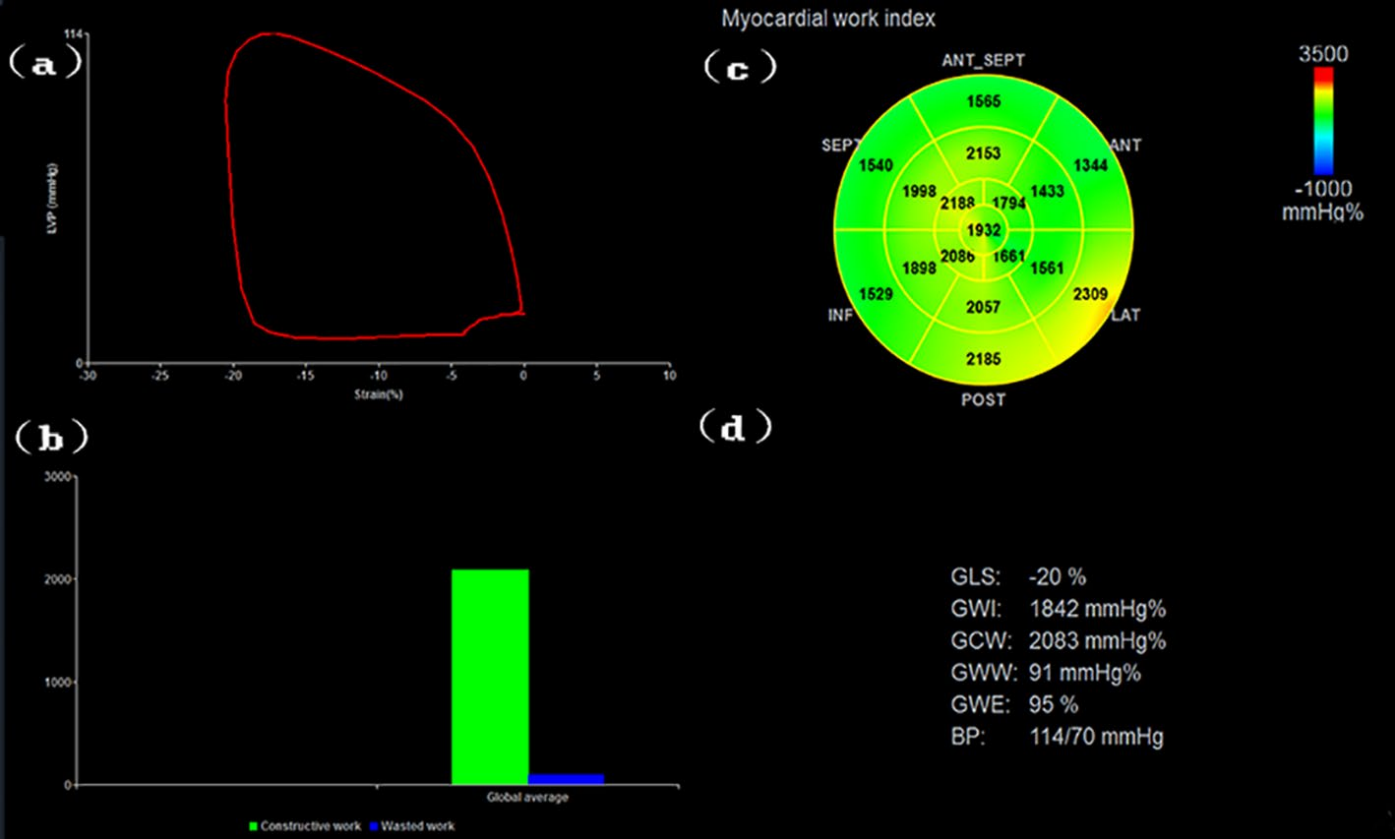
Schematic diagram of the NIMWI measurement results. **a** Changes in systolic blood pressure measured by the cuff as a function of the global longitudinal strain of the left ventricle. Red curve: overall pressure and strain of the left ventricle. The area under curve represents the global myocardial work index of the left ventricle. **b** Column chart of the global constructive work and waste work of the left ventricle. **c** Bull’s-eye diagram of the 17-segment myocardial work index of the left ventricle. **d** Results of the NIMWI parameters. NIMWI: non-invasive myocardial work index

The stored images were analysed offline by an Echo PAC BT203 (GE Vingmed Ultrasound, Horten, Norway) workstation. The durations of the LV isovolumic systole and ejection period were first determined according to the opening and closing of the aortic and mitral valves, and then the automatic functional imaging mode was selected. The endocardium of the LV long axis, four-chamber and two-chamber view were depicted from the level of the mitral valve annulus to the LV apex in sequence. The software automatically tracked the endocardium and epicardium of the left ventricle. The intracardiac and epicardial wrap were manually adjusted to ensure satisfactory tracking. Finally, Approve was clicked, and the blood pressure measured by the bronchial artery cuff was entered into the MW menu to obtain the LV pressure–strain loops (PSL), GLS and NIMWI. The NIMWI parameters included GWI, GCW, GWW and GWE, as shown in Fig. 4.

### Statistical analysis

All statistical analyses were performed using SPSS version 25.0 (SPSS Inc., IBM, Chicago, USA). Continuous variables were expressed as mean ± standard deviation if normally distributed. Independent-sample *t*-test was used to compare continuous variables between the uremic group and normal group. The Chi-squared test was used to compare count data. Correlation between continuous variables was carried out using Pearson’s or Spearman’s correlation coefficient as appropriate.

ROC curves were performed to determine the optimal sensitivity and specificity of MW parameter. The AUC was calculated to assess the performance of MW parameter for the detection impaired myocardium in uremic patients. According to the Youden index, the best threshold of each tested MW parameter was estimated. Ten subjects were randomly selected, and two observers used infra-class correlation coefficients to carry out repeatability tests for the myocardial work parameters GWI, GCW, GWW and GWE. A *P*-value < 0.05 was considered statistically significant.

## Data Availability

All data generated or analysed during this study are included in this published article.

## Abbreviations

NIMWI: Non-invasive myocardial work index
LV: Left ventricular
MW: Myocardial work
GWI: Global work index
GCW: Global constructive work
GWW: Global wasted work
GWE: Global work efficiency
ROC: Receiver operating characteristic
AUC: Area under the curve
CKD: Chronic kidney disease
LVEF: Left ventricular ejection fraction
GLS: Global longitudinal strain
2D: Two dimensional
SBP: Systolic blood pressures
DBP: Diastolic blood pressures
HR: Heart rate
BSA: Body surface area
IVSTd: Interventricular septum end-diastolic thickness
LVPWTd: Left ventricular posterior wall end-diastolic thickness
LVEDd: Left ventricular end-diastolic diameter
LVMI: Left ventricular mass index
ROC: Receiver operating characteristic
AUC: Area under the curve
DICOM: Digital Imaging and Communication in Medicine
PSL: Pressure–strain loops
